# Myocellular adaptations to short‐term weighted wheel‐running exercise are largely conserved during C26‐tumour induction in male and female mice

**DOI:** 10.1113/EP092504

**Published:** 2025-04-24

**Authors:** Stavroula Tsitkanou, Pieter Koopmans, Calvin Peterson, Ana Regina Cabrera, Ruqaiza Muhyudin, Francielly Morena, Sabin Khadgi, Eleanor R. Schrems, Tyrone A. Washington, Kevin A. Murach, Nicholas P. Greene

**Affiliations:** ^1^ Cachexia Research Laboratory, Exercise Science Research Center, Department of Health, Human Performance and Recreation University of Arkansas Fayetteville Arkansas USA; ^2^ Department of Neurology, Beth Israel Deaconess Medical Center Harvard Medical School Boston Massachusetts USA; ^3^ Molecular Muscle Mass Regulation Laboratory, Exercise Science Research Center, Department of Health, Human Performance and Recreation University of Arkansas Fayetteville Arkansas USA; ^4^ Cell and Molecular Biology Program University of Arkansas Fayetteville Arkansas USA; ^5^ Exercise Muscle Biology Laboratory, Exercise Science Research Center, Department of Health, Human Performance and Recreation University of Arkansas Fayetteville Arkansas USA

**Keywords:** catabolism, colorectal cancer, concurrent training, exercise training, muscle fibre‐type transition

## Abstract

This study investigated whether performing a translatable murine model of concurrent training after tumour induction affects adaptations in juvenile male and female tumour‐bearing mice. Male and female Balb/c mice were injected bilaterally with colon‐26 adenocarcinoma (C26) cells or PBS at 8 weeks of age. Half the mice then performed 24 days of voluntary wheel running with progressively increased load (PoWeR training), whereas the rest remained sedentary. Deuterium oxide‐based protein synthesis, muscle fibre‐type composition and size, protein turnover and mitochondrial markers were assessed 25 days after tumour induction. Average gastrocnemius muscle fibre size was smaller with PoWeR regardless of tumour in males and females, concomitant with a pronounced faster‐to‐slower fibre‐type transition. In male tumour‐bearing mice, PoWeR training resulted in greater *Redd1*, *Murf1* and *Pgc1α* mRNA content than all the other groups, along with lower overall running volume, food consumption and protein synthesis relative to control animals. Molecular measures followed a similar pattern in tumour‐bearing female mice with PoWeR, but food consumption, running volume and muscle protein synthesis were maintained. PoWeR training lowered gonadal fat during cancer cachexia in both sexes, and greater heart weight was observed regardless of tumour presence. A negative correlation was found between tumour weight and running distance. Collectively, PoWeR has a similar effect on muscle cellular phenotype in both sexes regardless of tumour presence, and a training effect in male mice with cancer cachexia was present despite molecular and protein synthesis dysregulation.

## Abstract (highlights)

- **What is the central question of this study?**
  Does short‐term concurrent resistance and endurance training (PoWeR) after inoculation of colon‐26 adenocarcinoma cells induce similar skeletal muscle adaptations in male and female mice?
- **What is the main finding and its importance?**
  Gastrocnemius muscle responses to short‐term exercise training were maintained in both sexes irrespective of the presence of tumour. Adaptations persisted despite a large tumour burden and cancer‐associated detriments that were most apparent in male mice. These findings might help to inform personalized exercise prescription to help treat cancer cachexia, a condition that lacks effective countermeasures.

## 1 INTRODUCTION

Cancer cachexia (CC) is a multifactorial and, so far, irreversible syndrome characterized by unintentional loss of body weight, mainly driven by losses of muscle and adipose mass in patients with cancer (Argiles et al., 2014). Up to 80% of cancer patients experience some degree of cachexia, and CC is responsible for 20%–40% of cancer‐related deaths (Argiles et al., 2014; Fearon et al., 2011). To date, there is no effective treatment to counteract the muscle wasting induced by cancer. Exercise training has been suggested as a potential non‐pharmacological therapeutic approach to attenuate or reverse CC (Courneya et al., 2000; Lira et al., 2014; Tsitkanou et al., 2022). The majority of clinical and preclinical studies have shown a beneficial impact of exercise in CC by decelerating disease progression and mitigating muscle wasting (Ballaro et al., 2019; Lira et al., 2014; Puppa et al., 2012; Ranjbar et al., 2019; Tsitkanou et al., 2022), with some others highlighting that exercise might be detrimental to muscle health and integrity owing to CC comorbidities (Argiles et al., 2012; Pin et al., 2015). Specifically, in the colon‐26 adenocarcinoma (C26) mouse model of colorectal cancer, endurance training does not prevent body weight and muscle loss, and even worsened the condition of the mice in the early stages of CC (i.e., 2 weeks after C26 inoculation) (Pin et al., 2015). Overall, the effectiveness of exercise training approaches as therapeutics during cancer remains an open question.

Concurrent resistance and endurance training at moderate intensities has been suggested as the optimal approach to exercise in CC (Ballaro et al., 2019; Ranjbar et al., 2019; Tsitkanou et al., 2022; Wolin et al., 2012). This recommendation is also based on guidelines by the American College of Sports Medicine (Wolin et al., 2012). A previous study using concurrent resistance (inclined ladder climbing with gradually increased resistance load) and aerobic (25 min wheel running at 5–9 m/min speed) training for 5.5 weeks (4 days/week) in male C26 tumour‐bearing mice prevented tumour‐induced muscle wasting and weakness (Ranjbar et al., 2019). The exercise‐induced prevention of CC was accompanied by a mitigated autophagy induction in tumour‐bearing mice, in addition to reverting muscle succinate dehydrogenase activity to healthy control levels (Ranjbar et al., 2019). A different form of concurrent training, including running on a motorized wheel with increased resistance at a moderate speed (5–11 m/min) for 15–45 min for 17 days (3–7 days/week), attenuated tumour‐induced muscle wasting and weakness. These beneficial effects appeared to be mediated by reduced reactive oxygen species and autophagy in male C26 tumour‐bearing mice (Ballaro et al., 2019). Likewise, long‐term wheel running with progressively increased resistance preserved muscle mass in a rhabdomyosarcoma mouse model, while partly reverting the cancer‐associated inflammatory and fibrotic transcriptome (Collao et al., 2023).

Mounting evidence suggests biological sexual dimorphism not only in the phenotype of CC, but also in the underlying molecular pathways associated with CC (Cabrera et al., 2023; Morena et al., 2024; Zhong & Zimmers, 2020). From a preclinical perspective, female Lewis Lung Carcinoma (LLC) and C26 tumour‐bearing mice show delayed development of muscle decrements, including losses in muscle myogenic potential, protein synthesis and mitochondrial function, compared with their male counterparts (Brown et al., 2017; Cabrera et al., 2023; Lim et al., 2022). In addition, the effectiveness of therapeutic interventions during CC might differ between males and females. We recently showed that administration of SkQ1 [10‐(6'‐plastoquinonyl) decyltriphenylphosphonium], which targets mitochondria‐derived oxidative stress, attenuates muscle wasting induced by C26 tumours in male, but not female, C26 tumour‐bearing mice (Tsitkanou et al., 2024). Considering the possibility of biological sex divergence in therapeutic responsiveness, it becomes crucial to determine whether this effect persists with exercise. Clinical studies have provided initial evidence that exercise is an efficient therapeutic intervention for attenuating muscle wasting and muscle strength loss in both biological sexes (Adraskela et al., 2017; Galvao et al., 2010). Indirect and observational comparisons between preclinical studies featuring different biological sexes suggest that exercise‐induced adaptations in skeletal muscle are similar in female and male tumour‐bearing mice (Hardee et al., 2016; Sato et al., 2019). Specifically, a representative form of rodent resistance training involving lower‐limb contractions induced by high‐frequency electric stimulation increased muscle mass and fibre size (type IIa and IIb) in the tibialis anterior (TA) muscle and improved protein synthesis and skeletal muscle oxidative capacity similarly in female (Sato et al., 2019) and male (Hardee et al., 2016) *Apc^Min/+^* mice.

Considering the potential viability of exercise as a preventive therapeutic, in this study we aimed to investigate whether performing short‐term concurrent resistance and aerobic training after inoculation of C26 cells would decelerate CC progression and induce different skeletal muscle adaptations in juvenile male and female mice. We used a promising translatable murine model of concurrent training, known as PoWeR training (Murach et al., 2019; Murach, McCarthy et al., 2020), which, to our knowledge, has not been used previously in studies of colon cancer‐induced cachexia. Our approach clinically aims to mimic the initiation of an exercise programme upon initial cancer detection or tumour development as a CC preventative. We hypothesized that CC would not impede concurrent exercise training adaptations and that concurrent exercise training might decelerate CC development and progression potentially via distinct pathways in males and females.

## 2 MATERIALS AND METHODS

### 2.1 Ethics approval

This study was approved by the University of Arkansas – Fayetteville IACUC (AUP 22035) in compliance with the Animal Welfare Act, The Public Health Service Policy on Humane Care and Use of Laboratory Animals, and our Animal Welfare Assurance. The University's IACUC programme is accredited by the Association for Assessment and Accreditation of Laboratory Animal Care International (AAALAC) and provides the utmost care for laboratory animals. We understand the ethical principles under which the journal operates, and our work complies with the journal's animal ethics checklist.

### 2.2 Animal interventions

All animal methods were approved by the Institutional Animal Care and Use Committee of the University of Arkansas (AUP#220035). Male (*n* = 37) and female (*n* = 35) Balb/c mice were purchased from the Jackson Laboratory (Bar Harbor, ME, USA; stock no. 001026) and housed in a temperature‐controlled environment following a 12 h–12 h light–dark cycle, at ∼22°C. All mice had ad libitum access to normal rodent chow and water throughout the study. At 8 weeks of age, all mice were subcutaneously and bilaterally injected with either colon‐26 carcinoma (C26; National Cancer Institute) cells (total cell volume of 1 × 10^6^) suspended in 200 µL of sterile PBS (i.e., 5 × 10^5^ cells suspended in 100 µL of sterile PBS for each flank) or an equal volume of sterile PBS as a sham control (Bonetto et al., 2016), while they were under general anaesthesia with a mixture of 2% isoflurane and oxygen (0.5 L/min). Tumours were allowed to develop for 25 days, which was considered the humane end‐point of the study based on recent studies with the model (Cabrera et al., 2023; Delfinis et al., 2022).

**FIGURE 1:**
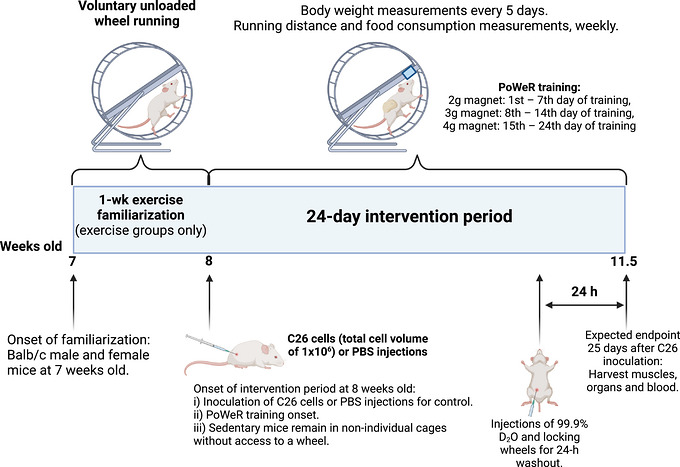
Experimental design of the study.

A week before tumour implantation with C26 cells or PBS, half of the mice were randomly assigned to exercise groups and single‐housed in a wheel‐running cage with an unloaded wheel (i.e., without resistance) for familiarization. Immediately after the C26/PBS implants, these mice started performing a 24 day PoWeR training regimen (Murach et al., 2019; Murach, McCarthy et al., 2020; Figure 1). PoWeR training, being entirely voluntary, serves as an ideal model to use during the stress of tumour inoculation, aligning with the principle of ‘refinement’ from the ‘3 Rs’ of animal research, with a focus on enhancing animal welfare. A short‐term version of the PoWeR training (i.e., 3.5 weeks instead of 8 weeks) was used here, because 24 days after tumour induction is the maximum duration of tumour development in C26 tumour‐bearing mice prior to reaching a humane end‐point and, as such, the longest we could power‐train our mice without presenting welfare concerns. Running distances (in kilometres per day) were recorded using Clocklab software (Actimetrics, Wilmette, IL, USA).

The PoWeR training protocol used in this study consisted of voluntary wheel running with progressively increased loading from 2 to 4 g of weight (i.e., 2 g during the 1st–7th day of training, 3 g during the 8th–14th day of training, and 4 g during the 15th–24th day of training). The rest of the mice remained sedentary, without wheel access, throughout the study, in group housing. Therefore, four experimental groups per sex (eight groups total) were included in this study: (1) sedentary mice injected with PBS (PBS SED; *n* = 11 males and *n* = 10 females); (2) mice injected with PBS that performed PoWeR training (PBS PoWeR; *n* = 9 males and *n* = 9 females); (3) sedentary mice injected with C26 cells (C26 SED; *n* = 9 males and *n* = 9 females); and (4) mice injected with C26 cells that performed PoWeR training (C26 PoWeR; *n* = 8 males and *n* = 7 females).

Body weight was measured every 5 days (Figure S1). Food consumption (in grams per day) and running distance (in kilometres per day) were monitored weekly. The area under the curve (AUC) was calculated including the weekly measurements (1st to the 4th week of exercise) in running distance and food consumption. Twenty‐four days after the C26 and PBS injections, intraperitoneal injections with 99.9% deuterium oxide (D_2_O) were performed (see Section 2.4), after which the wheels in wheel‐running cages were locked as a washout/recovery from exercise training 24 h before harvesting tissues (Figure 1).

At the end‐point (i.e., 25 days post C26 inoculation) skeletal muscle tissues from hindlimbs and forelimbs, organs (liver, spleen and heart), gonadal fat, tumours and blood were collected, weighed, snap frozen and stored at −80°C for future analysis. To maintain sample integrity for protein synthesis assays, muscles and blood were collected under isoflurane anaesthesia [mixture of 2% isoflurane and oxygen (0.5 L/min)], after which the mice were killed by cervical dislocation prior to completion of tissue collection. Tibia length was measured as a surrogate of body size index. Tibia lengths were not statistically different between groups within each biological sex. Although it was not feasible to blind investigators to subcutaneous tumours or wheel access during experimental interventions, all downstream analyses were performed in a blinded manner wherever possible.

### 2.3 RNA extraction and mRNA analysis

Gastrocnemius muscle was used for the mRNA analysis as the most affected muscle by the PoWeR training regimen of this study. RNA extraction, complementary DNA synthesis and quantitative real‐time PCR using the Real‐Time PCR system (Applied Biosystems, MA, USA) were performed as described by members of our laboratory (Cabrera et al., 2023; Lim et al., 2022; Tsitkanou et al., 2024). RNA gene expression of 18S (Applied Biosystems, ID Mm03928990_g1) was used as the housekeeping gene reaction. In addition, we used the following TaqMan probes: *Atrogin1* (*Fbxo32*; Mm00499523_m1), *Murf1* (*Trim63*; Mm01185221_m1), *Ubc* (Mm02525934_g1), *Gadd45a* (Mm00432802_m1), *Deptor* (Mm01195339_m1), *Redd1* (*Ddit4*; Mm00512504_g1), *Opa1* (Mm01349707_g1), *Bnip3* (Mm01275600_g1), *Mapk14* (*p38a*; Mm01301009_m1), *Pgc1a* (Mm00447183_m1), *Tnf‐alpha* (Mm00443258_m1) and *Il‐6* (Mm00446190_m1). Final quantification of mRNA levels was calculated through the ΔΔ*Ct* method using 18S as the loading control. The 18S *Ct* value did not differ between the experimental groups. Relative quantification was calculated using the 2^−ΔΔ^ *^Ct^* method (Livak & Schmittgen, 2001), as described in our previous studies (Cabrera et al., 2023; Greene et al., 2015; Tsitkanou et al., 2024). Fold changes were calculated by dividing the 2^−ΔΔ^ *^Ct^* of each sample by the average 2^−ΔΔ^ *^Ct^* of the healthy control group. For statistical analysis, we used the fold change values.

### 2.4 Deuterium administration and 24 h fractional protein synthetic rate

Mice were injected intraperitoneally with 99.9% deuterium oxide (D_2_O) (20 µL/g of body weight; catalogue no. 151882‐1L, Millipore Sigma) 24 h before tissue collection (Cabrera et al., 2023; Lim et al., 2022; Tsitkanou et al., 2024). After injections, drinking water was supplemented with 4% D_2_O to maintain plasma enrichment (Gasier et al., 2009). The fractional synthetic rate (FSR) was measured in gastrocnemius, using gas chromatography–mass spectrometry (GCMS; 7890A and 5977A; Agilent, USA), as described previously by members of our laboratory (Brown et al., 2017; Cabrera et al., 2023; Lim et al., 2022; Tsitkanou et al., 2024). All data were normalized by deuterated plasma enrichment and analysed via enrichment ratio as described (Cabrera et al., 2023; Lim et al., 2022; Tsitkanou et al., 2024).

### 2.5 Immunohistochemistry

At tissue collection, gastrocnemius and soleus muscles were mounted in optimal cutting temperature (OCT) medium and snap‐frozen in liquid nitrogen‐cooled isopentane, then stored at −80°C until later analyses. Fibre cross‐sectional area (CSA) and myosin heavy chain (MyHC) fibre‐type analyses on the gastrocnemius and soleus were performed as described by Murach, Mobley et al. (2020). Briefly, 8‐µm‐thick sections were cut at the mid‐belly using a cryostat and allowed to air dry for ≥1 h. Sections were then encircled in a hydrophobic Polymerase Activity Probe pen barrier (ImmEdge; Vector Laboratories) and incubated with primary antibodies for dystrophin (1:100, ab15277; Abcam, St. Louis, MO, USA) and MyHCs I, IIA and/or IIB (1:100; BA‐D5, SC‐71 and BF‐F3; Developmental Studies Hybridoma Bank, Iowa City, IA, USA) overnight in a PBS cocktail at 4°C. MyHC IIX was left unstained. After PBS washes, slides were incubated in a PBS cocktail of isotype‐specific secondary antibodies conjugated to different fluorescent tags at room temperature for 60–90 min (1:200; AF555, catalogue no. 21426; AF488, catalogue no. 21121; AF647, catalogue no. 21242; AF568, catalogue no. A11011; Invitrogen, ThermoFisher Scientific, Waltham, MA, USA). Following PBS washes, 4′,6‐diamidino‐2‐phenylindole (DAPI; 1:10 000; catalogue no. D1306; Invitrogen) was applied, after which slides were mounted using a 50:50 solution of PBS and glycerol.

### 2.6 Image capture and analyses

For immunohistochemistry, all images were captured as whole‐muscle cross‐sections at ×20 magnification using a Zeiss AxioImager M2. Fibre CSA and fibre‐type distribution were analysed using MyoVision, as described (Viggars et al., 2022; Wen et al., 2018). An average of 6187 ± 1326 and 824 ± 140 fibres (per mouse) were analysed in the gastrocnemius and soleus, respectively. Central myonuclei were counted manually in Zen by a blinded, trained technician and considered central if there was a discernable distance between DAPI and the dystrophin cell border. Muscle spindle size, expressed as CSA (in micrometres squared), was analysed as a marker of muscle quality and identified by a thick basement membrane (muscle spindle capsule) that surrounds the intrafusal fibres. Spindles were traced manually in Zen for CSA analysis. It is worth noting that not all cross‐sections presented a muscle spindle, hence sample sizes are lower for this analysis (*n* = 3–5 per group). Central myonuclei and spindle size were analysed only in soleus muscle (Figure S2).

### 2.7 Statistics

All data are presented as the mean ± SD. A two‐way ANOVA within each sex, with factors of tumour (C26 vs. PBS) or PoWeR intervention (PoWeR vs. SED) was used as the global analysis for each dependent variable of interest. Tukey's *post hoc* analysis was used to examine pairwise differences whenever a significant interaction was found. Student's unpaired *t*‐test comparisons were made to assess the differences in running distance and food consumption between sedentary tumour‐bearing mice (C26 SED) and exercised tumour‐bearing mice (C26 PoWeR). On that note, food consumption per mouse could not be measured accurately in sedentary groups because mice in sedentary conditions were group housed. Thus, we did not have as many replicates for statistical analysis (*n* = 2–3 cages per sedentary condition). However, representative bars/lines have been added in the figures. A one‐way ANOVA accompanied by Tukey's *post hoc* analysis was used to determine whether there were significant differences between exercised non‐tumour‐bearing mice (PBS PoWeR), C26 SED and C26 PoWeR in muscle and organ weights expressed as the percentage difference from the healthy control group (PBS SED). Pearson's correlation (*r*) was used to assess the relationships between running distance (using AUC values) and muscle/tumour weights in exercised groups, and between *Pgc1α* mRNA content and central myonuclei. To assess biological sex differences in key variables (including body weight, tissue weights, running distance, food consumption and protein synthesis) directly, we performed a two‐way ANOVA between biological sexes using percentage differences from same‐sex healthy control animals as the baseline for each (tissue weights and protein synthesis) or percentage differences from the day of C26 inoculation (tumour‐free body weight) or AUC (running distance and food consumption). Additionally, we used Student's unpaired *t*‐test to compare males and females in the C26 PoWeR groups for the percentage change in food consumption from the first week of intervention to the end‐point (see Table S1 and Figures S3 and S4). A value of *P* ≤ 0.05 was established a priori as the level of significance. All statistical analysis was performed using GraphPad Prism v.10.1.2 (GraphPad Software, Boston, MA, USA). Figures were created with GraphPad or BioRender.

## 3 RESULTS

### 3.1 Tumour decreases body weight, gonadal fat and muscle mass, while exercise adaptations were similar in both males and females irrespective of tumour

**FIGURE 2:**
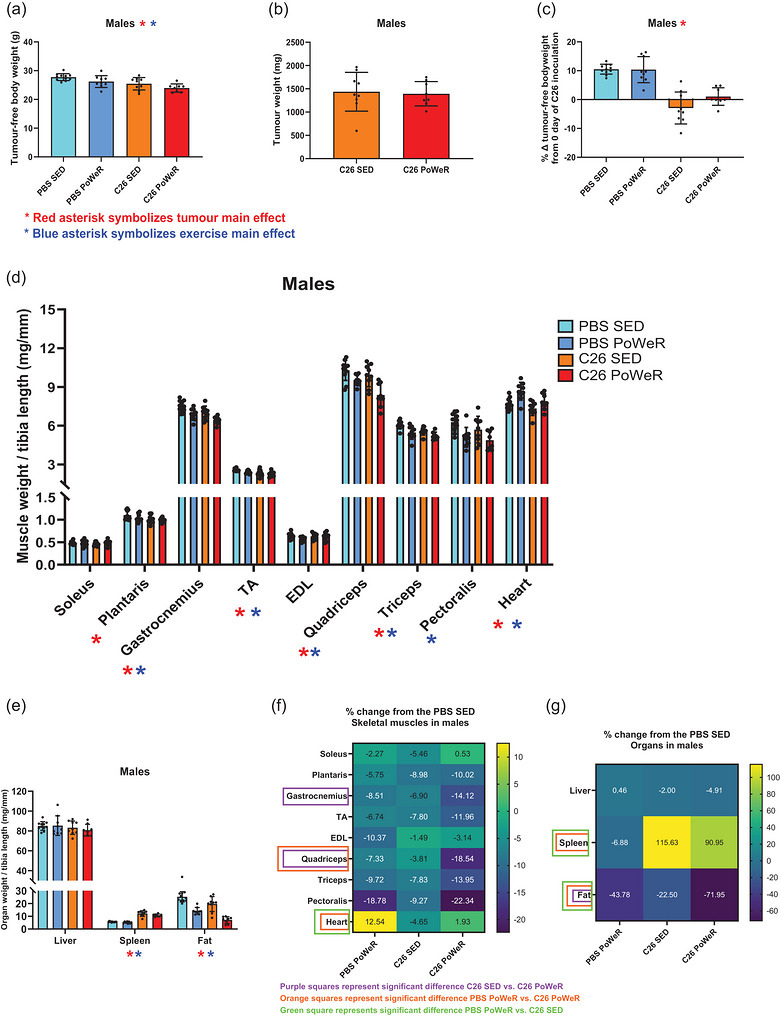
Four‐week PoWeR training further increases cancer‐induced muscle wasting and fat loss in males. (a,b) Tumour‐free body weight (in grams; a) and tumour weight (in milligrams; b) at the experimental end‐point. (c) Percentage difference in tumour‐free body weight at the experimental end‐point from the baseline body weight on the day of inoculation of C26 cells. (d,e) Muscle weights normalized by tibia length (in milligrams per millimetre) of soleus, plantaris, gastrocnemius, tibialis anterior (TA), extensor digitorum longus (EDL), quadriceps and heart (d), in addition to organ weights normalized by tibia length (in milligrams per millimetre) of liver, spleen and fat (e) at the experimental end‐point (25 days after the inoculation of C26 cells) in males. (f,g) Heatmaps of the percentage difference of PBS PoWeR, C26 SED and C26 PoWeR from the healthy control group (PBS SED) in skeletal muscle tissues (f) and organs in males. Red asterisks symbolize a significant (*p* < 0.05) tumour main effect (C26 vs. PBS), and blue asterisks symbolize a significant (*p* < 0.05) PoWeR exercise main effect (PoWeR vs. SED) after performing two‐way ANOVA. Purple, orange and green squares (f,g), symbolize significant differences (*p* < 0.05) between: C26 SED versus C26 PoWeR (purple), PBS PoWeR versus C26 PoWeR (orange) and PBS PoWeR versus C26 SED (green), after performing unpaired one‐way ANOVA accompanied by Tukey's *post hoc* analysis.

**FIGURE 3:**
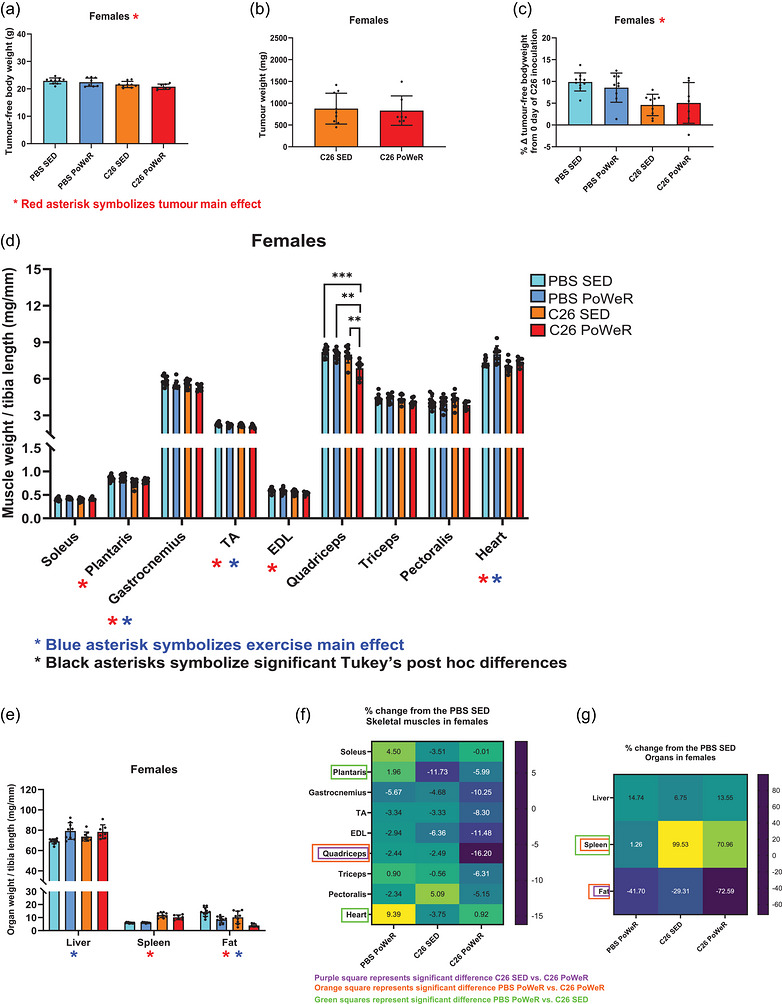
As for males, 4 weeks of PoWeR training increases cancer‐induced muscle wasting and fat loss in females. (a,b) Tumour‐free body weight (in grams; a) and tumour weight (in milligrams; b) at the experimental end‐point. (c) Percentage difference in tumour‐free body weight at the experimental end‐point from the baseline body weight on the day of inoculation of C26 cells. (d,e) Muscle weights normalized by tibia length (in milligrams per millimetre) of soleus, plantaris, gastrocnemius, tibialis anterior (TA), extensor digitorum longus (EDL), quadriceps and heart (d), in addition to organ weights normalized by tibia length (in milligrams per millimetre) of liver, spleen and fat (e) at the experimental end‐point (25 days after the inoculation of C26 cells) in females. (f,g) Heatmaps of the percentage difference of C26 SED and C26 PoWeR from the healthy control group (PBS SED) in skeletal muscle tissues (f) and organs (g) in females. Red asterisks symbolize a significant (*p* < 0.05) tumour main effect (C26 vs. PBS), and blue asterisks symbolize a significant (*p* < 0.05) PoWeR exercise main effect (PoWeR vs. SED) after performing two‐way ANOVA. Black asterisks symbolize significant (^**^ *p* < 0.01 and ^***^ *p* < 0.001) Tukey's *post hoc* differences, after confirming a significant interaction between tumour and exercise PoWeR intervention. Purple, orange and green squares (f,g), symbolize significant differences (*P* < 0.05) between C26 SED versus C26 PoWeR (purple), PBS PoWeR versus C26 PoWeR (orange) and PBS PoWeR versus C26 SED (green), after performing unpaired one‐way ANOVA accompanied by Tukey's *post hoc* analysis.

Tumour‐bearing mice had lower tumour‐free body weight (in grams) at the experimental end‐point (i.e., 25 days after C26 inoculation) and percentage change of the end‐point tumour‐free body weight from initial body weight (measured on the day of C26 inoculation; as a percentage) in both males (*p* = 0.0004, Figure 2a; and *p* < 0.0001, Figure 2c, respectively) and females (*p* = 0.0009, Figure 3a; and *p* = 0.0003, Figure 3c, respectively). Only in males, exercise affected tumour‐free body weight, with exercised mice presenting lower tumour‐free body weight at the experimental end‐point compared with sedentary mice (*p* = 0.013, Figure 2a). Exercise did not affect tumour weights in either males (Figure 2b) or females (Figure 3b). Tumour‐bearing mice had lower muscle weights normalized by tibia length (in milligrams per millimetre) in plantaris (*p* = 0.0164 in males; *p* = 0.0011 in females), gastrocnemius (*p* = 0.0027 in males; *p* = 0.0307 in females), TA (*p* = 0.0083 in males; *p* = 0.0427 in females), extensor digitorum longus (EDL; *p* = 0.0224 in females only), quadriceps (*p* = 0.005 in males; *p* = 0.0015 in females) and triceps (*p* = 0.0045 in males only), compared with non‐tumour‐bearing mice (Figures 2d and 3d). Exercised mice also presented lower muscle weights in gastrocnemius (*p* = 0.0003 in males; *p* = 0.0096 in females), TA (*p* = 0.0254 in males; *p* = 0.0474 in females), quadriceps (*p* = 0.0001 in males; *p* = 0.0016 in females), triceps (*P* = 0.0003 in males only) and pectoralis (*p* = 0.0007 in males only) compared with sedentary sex‐controlled mice (Figures 2d and 3d). Heart weight was lower in tumour‐bearing mice than in non‐tumour‐bearing mice (tumour main effect: *p* = 0.0041 in males; *p* = 0.0115 in females), but greater in exercised mice than in sedentary mice (exercise main effect: *p* = 0.0005 in males; *p* = 0.0043 in females), although no interaction was found between tumour × exercise training in either biological sex (Figures 2d and 3d). An interaction between tumour × exercise training in muscle weights was found only in quadriceps of females (*p* = 0.0219), where the quadriceps weight of exercised tumour‐bearing mice was lower compared with every other group (i.e., C26 PoWeR vs. C26 SED, *p* = 0.0021; C26 PoWeR vs. PBS PoWeR, *p* = 0.0021; C26 PoWeR vs. PBS SED, *p* = 0.0002; Figure 3d). Furthermore, tumour‐bearing mice had higher spleen weight (*p* < 0.0001 in males; *p* < 0.0001 in females) and lower gonadal fat weight (*p* < 0.0001 in males; *p* = 0.0006 in females) compared with non‐tumour‐bearing mice, and exercised mice presented lower spleen weight (*p* = 0.0341 in males only) and gonadal fat weight (*p* < 0.0001 in males; *p* < 0.0001 in females) and higher liver weight (*p* = 0.0007 in females only) compared with sedentary mice (Figures 2e and 3e).

We also assessed the phenotype as a percentage difference from the healthy control group (PBS SED), followed by a subsequent one‐way ANOVA accompanied by Tukey's *post hoc* analysis (PBS PoWeR vs. C26 SED vs. C26 PoWeR). A significant difference between C26 SED and C26 PoWeR was found, with exercised tumour‐bearing mice having a larger difference in gastrocnemius (−7% vs. −14%, *p* = 0.0478 in males), quadriceps (−4% vs. −19%, *p* = 0.0014 in males; −2% vs. −16%, *p* = 0.0042 in females) and fat (−23% vs. −72%, *p* < 0.0001 in males; −29% vs. −73%, *p* = 0.0032 in females) compared with sedentary tumour‐bearing mice (Figures 2f,g and 3f,g). Significant differences were also found between the two exercised groups (i.e., PBS PoWeR vs. C26 PoWeR) with exercised tumour‐bearing mice exhibiting a greater decrease in quadriceps (−7% vs. −19%, *p* = 0.0142 in males; −2% vs. −16%, *p* = 0.0041 in females), lower increase in heart (13% vs. 2%, *p* = 0.0347 in males), lower decrease in spleen (−7% vs. 91%, *p* < 0.0001 in males; 1% vs. 71%, *p* < 0.0001 in females) and greater decrease in fat (−44% vs. −72%, *p* = 0.004 in males; −42% vs. −73%, *p* = 0.0363 in females) compared with exercised non‐tumour‐bearing mice (Figures 2f,g and 3f,g). Finally, compared with exercised non‐tumour‐bearing mice, sedentary tumour‐bearing mice presented significantly lower (PBS PoWeR vs. C26 SED) plantaris (2% vs. −12%, *p* = 0.0058 in females) and heart weights (13% vs. −5%, *p* = 0.0005 in males; 9% vs. −4%, *p* = 0.0029 in females), in addition to higher spleen (−7% vs. 116%, *p* < 0.0001 in males; 1% vs. 100%, *p* < 0.0001 in females) and fat weights (−44% vs. −23%, *p* = 0.0255 in males) (Figures 2f,g and 3f,g). Direct comparisons of biological sex based on the percentage difference from same‐sex healthy control animals (tissue weights) or the day of C26 inoculation (tumour‐free body weight) can be found in Table S1 and Figure S3.

### 3.2 Tumour decreases running distance and food consumption only in males

**FIGURE 4:**
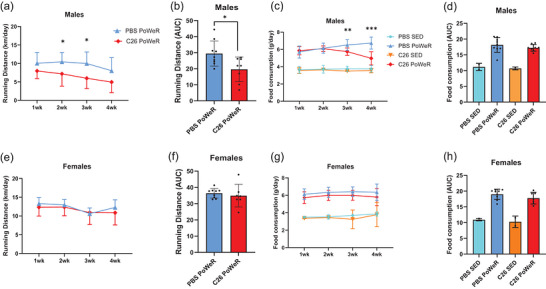
Running distance (a,b,e,f) and food consumption (c,d,g,h) decrease during progression of cancer cachexia (CC) in exercised male mice but not in exercised female mice. Running distance and food consumption are analysed per week (from the first to fourth week of training; a,c,e,g) and as the area under the curve (AUC; b,d,f,h). Food consumption per mouse could not be measured accurately in sedentary groups because mice in sedentary conditions were group housed. Thus, there were not as many replicates for statistical analysis (*n* = 2–3 cages per sedentary condition). However, representative bars/lines for the sedentary groups (i.e., PBS SED and C26 SED) have been added (c,d,g,h). Black asterisks symbolize a significant (*p* < 0.05) difference in PBS PoWeR versus C26 PoWeR after performing Student's unpaired *t*‐test.

Tumour affected running performance only in males, with exercised tumour‐bearing mice presenting lower daily running distance during the second (*p* = 0.0367) and third weeks (*p* = 0.0144) of training compared with exercised non‐tumour‐bearing mice (Figure 4a). Likewise, food consumption in males was lower in exercised tumour‐bearing mice during the third (*p* = 0.0096) and fourth (*p* = 0.0003) weeks of training compared with exercised non‐tumour‐bearing mice (Figure 4c). The AUC in running distance was also lower in exercised tumour‐bearing mice compared with exercised non‐tumour‐bearing mice (*p* = 0.0191; Figure 4b), but no difference was found in the AUC in food consumption (Figure 4d). In females, tumour did not affect running performance or food consumption in exercised mice (Figure 4e–h). A direct comparison of biological sex on running distance and food consumption can be found in Figure S4.

### 3.3 Running distance is inversely related to muscle and tumour weight in male and female mice

**FIGURE 5:**
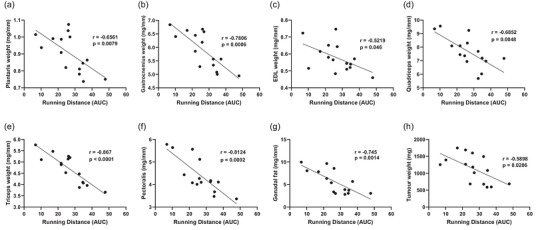
Running distance is negatively correlated with cancer‐induced muscle and fat loss and with tumour weight at the experimental end‐point. Pearson's *r* correlation analysis showed that normalized weight (by tibia length) of plantaris (a), gastrocnemius (b), extensor digitorum longus (EDL; c), quadriceps (d), triceps (e), pectoralis (f), gonadal fat (g) and tumour weight (h) are negatively associated (*p* < 0.05) with the area under the curve (AUC) from the 4 week running distance of exercised tumour‐bearing male and female mice.

Pearson's correlations were performed to assess whether running distance was related to the degree of tumour‐induced muscle wasting and tumour growth. Specifically, exercised tumour‐bearing male and female mice were pooled together for Pearson's *r* analysis (*n* = 15). Although exercise did not affect tumour weights in either males (Figure 2b) or females (Figure 3b), running distance, expressed as the AUC, was negatively associated with masses of plantaris (*r* = −0.6561, *p* = 0.0079), gastrocnemius (*r* = −0.7806, *p* = 0.0006), EDL (*r* = −0.5219, *p* = 0.046), quadriceps (*r* = −0.6852, *p* = 0.0048), triceps (*r* = −0.867, *p* < 0.0001), pectoralis (*r* = −0.8124, *p* = 0.0002), gonadal fat (*r* = −0.745, *p* = 0.0014) and tumour (*r* = −0.5898, *p* = 0.0206) weights (Figure 5a–h). Interestingly, similar results were found irrespective of tumour presence. Specifically, running distance was negatively associated with muscle weights either when exercised tumour‐bearing and non‐tumour‐bearing male and female mice were pooled together (*n* = 33) or when only exercised non‐tumour‐bearing male and female mice were pooled together (*n* = 18) (Table S2).

### 3.4 Exercise‐induced muscle mass loss in males and females might be explained by negative protein turnover

**FIGURE 6:**
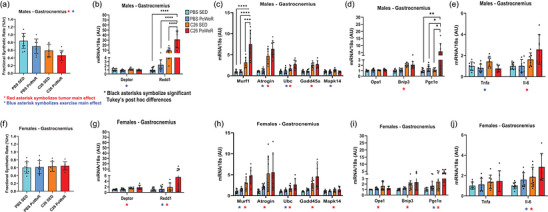
PoWeR exercise training decreases protein synthesis in gastrocnemius in males but not in females, and both biological sexes exhibit an exercise‐induced dysregulation in anabolic repressors and catabolic markers. (a,f) Fractional synthetic rate (as a percentage per hour) in gastrocnemius of males (a) and females (f). (b–e,g–j) Gene expression (normalized by 18S housekeeping gene) of anabolic repressors (*Deptor* and *Redd1*), catabolic markers (*Murf1*, *Atrogin*, *Ubc*, *Gadd45a* and *Mapk14*), mitochondrial markers (*Opa1*, *Bnip3* and *Pgc1a*) and inflammatory markers (*Tnfa* and *Il‐6*) in gastrocnemius of males (b–e) and females (g–j). Red asterisks symbolize a significant (*p* < 0.05) tumour main effect (C26 vs. PBS), blue asterisks symbolize a significant (*p* < 0.05) PoWeR exercise main effect (PoWeR vs. SED), and black asterisks symbolize significant (^*^ *p* < 0.05, ^**^ *p* < 0.01, ^***^ *p* < 0.001 and ^****^ *p* < 0.0001) Tukey's *post hoc* differences, after confirming a significant interaction between tumour and exercise PoWeR intervention.

In males, significant main effects of tumour (*p* = 0.0001) and exercise (*p* = 0.0242) were found in gastrocnemius FSR, with tumour‐bearing mice presenting lower FSR than non‐tumour‐bearing mice and exercised mice presenting lower mixed muscle FSR than non‐exercised mice (Figure 6a). A direct comparison of FSR differences from healthy control between sexes is in Figure S3. To assess the potential molecular mechanisms contributing to lower muscle protein synthesis in male mice induced by exercise, the mRNA content of anabolic repressors (*Deptor* and *Redd1*) and catabolic markers (*Murf1*, *Atrogin*, *Ubc*, *Gadd45a* and *Mapk14*) were measured in gastrocnemius muscle. In comparison to sedentary mice, the mRNA content of *Deptor* (*p* = 0.0267) was lower in male exercised mice (Figure 6b), and a significant interaction between tumour and exercise was found in the mRNA content of *Redd1* (*p* = 0.0012) in males, with exercised tumour‐bearing mice presenting higher *Redd1* levels (C26 PoWeR vs. C26 SED, *p* < 0.0001; C26 PoWeR vs. PBS PoWeR, *p* < 0.0001; C26 PoWeR vs. PBS SED, *p* < 0.0001) compared with every other group (Figure 6b). Compared with non‐tumour‐bearing mice, the mRNA content of *Atrogin* (*p* < 0.0001), *Ubc* (*p* < 0.0001) and *Gadd45a* (*p* < 0.0001) was higher in male tumour‐bearing mice (Figure 6c). In comparison to sedentary mice, the mRNA content of *Atrogin* (*p* = 0.0408), *Ubc* (*p* = 0.014) and *Mapk14* (*p* = 0.0204) was higher in male exercised mice (Figure 6c). A significant interaction between tumour and exercise was found in the mRNA content of *Murf1* (*p* = 0.0015) in males, with exercised tumour‐bearing mice presenting higher *Murf1* levels (C26 PoWeR vs. C26 SED, *p* = 0.0002; C26 PoWeR vs. PBS PoWeR, *p* < 0.0001; C26 PoWeR vs. PBS SED, *p* < 0.0001) compared with every other group (Figure 6c). Mitochondrial (*Bnip3* and *Pgc1a*) and inflammatory (*Tnfa* and *Il‐6*) markers were also affected by tumour and/or exercise in males (Figure 6d,e). Specifically, tumour‐bearing males mice had a higher mRNA content of *Bnip3* (*p* < 0.0001) and *Il‐6* (*p* = 0.0004) compared with non‐tumour‐bearing mice. Also, exercised male mice presented lower mRNA content of *Tnfa* (*p* = 0.0062) compared with sedentary male mice (Figure 6e). Interestingly, a significant interaction between tumour and exercise was found in the mRNA content of *Pgc1a* in males (*p* = 0.0424), with exercised tumour‐bearing mice presenting higher *Pgc1a* levels in comparison to every other group (C26 PoWeR vs. C26 SED, *p* = 0.0159; C26 PoWeR vs. PBS PoWeR, *p* = 0.0214; C26 PoWeR vs. PBS SED, *p* = 0.0057; Figure 6d).

In females, no main effect or interaction between tumour and exercise was found in gastrocnemius FSR (Figure 6f). Like males, in females the mRNA content of *Deptor* (*p* < 0.0001), *Redd1* (*p* = 0.0058), *Murf1* (*p* < 0.0001), *Atrogin* (*p* = 0.0004), *Ubc* (*p* = 0.0002), *Gadd45a* (*p* < 0.0001), *Mapk14* (*p* = 0.0243), *Opa1* (*p* = 0.0263), *Bnip3* (*p* = 0.0004), *Pgc1a* (*p* = 0.0001) and *Il‐6* (*p* = 0.0086) was higher in tumour‐bearing mice compared with non‐tumour‐bearing mice (Figure 6g–j). Furthermore, compared with sedentary mice, the mRNA content of *Redd1* (*p* = 0.0377), *Murf1* (*p* = 0.0067), *Ubc* (*p* = 0.0403), *Pgc1a* (*p* = 0.0108) and *Il‐6* (*p* = 0.0086) was higher in female exercised mice (Figure 6g–j). No interaction between tumour and exercise was found in any of the analysed anabolic repressors, catabolic, mitochondrial and inflammatory factors in females (Figure 6g–j).

### 3.5 Short‐term PoWeR training induces similar cellular changes in both biological sexes irrespective of tumour

**FIGURE 7:**
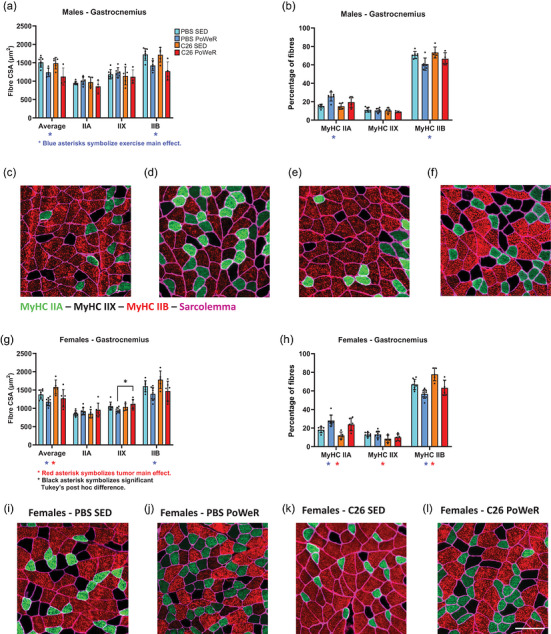
In gastrocnemius, PoWeR exercise training induces muscle fibre adaptations in both biological sexes. Exercised males and females have lower average cross‐sectional area (CSA) and CSA in myosin heavy chain (MyHC) type IIB (a and g, respectively), in addition to a higher percentage of MyHC IIA and lower percentage of MyHC IIB (b and h, respectively), in comparison to the sedentary mice. Red asterisks symbolize a significant (*p* < 0.05) tumour main effect (C26 vs. PBS), and blue asterisks symbolize a significant (*p* < 0.05) PoWeR exercise main effect (PoWeR vs. SED). The black asterisk symbolizes a significant (^*^ *p* < 0.05) Tukey's *post hoc* difference, after confirming a significant interaction between tumour and exercise PoWeR intervention. Immunofluorescence images (×20 magnification) are presented for male groups (c–f) and for female groups (i–l). MyHC IIA fibres are stained green, MyHC IIX fibres are unstained (‘black’ fibres), MyHC IIB fibres are stained red, and sarcolemma is stained purple.

Gastrocnemius was selected for analysis by immunohistochemistry as the major muscle with distinct MyHC compositions recruited during wheel running in rodents and characterized as responsive to PoWeR training (Murach, Mobley et al., 2020). Irrespective of tumour, both males and females exhibited lower average fibre CSA in gastrocnemius (*p* = 0.0002 in males; *p* = 0.0004 in females) with lower fast glycolytic MyHC IIB fibre CSA (*p* = 0.0002 in males; *p* = 0.0032 in females) after 24 days of PoWeR training, compared with sedentary mice (Figure 7a,g). Females also exhibited an interaction between PoWeR training and tumour (*p* = 0.0185; C26 PoWeR greater than PBS PoWeR, *p* = 0.013) in CSA of MyHC IIX fibres, in addition to a main effect of tumour on average fibre CSA (*p* = 0.0249; Figure 7g). Muscle fibre‐type composition was also altered in gastrocnemius, with exercised male and female mice having a higher percentage of MyHC IIA fibres (*p* = 0.0005 in males; *p* < 0.0001 in females) and lower percentage of MyHC IIB fibres (*p* = 0.0035 in males; *p* < 0.0001 in females) compared with sedentary mice (Figure 7b,h). In females, tumour‐bearing mice presented a lower percentage of MyHC IIA (*p* = 0.0166) and MyHC IIX (*p* = 0.0099) fibres and a higher percentage of MyHC IIB fibres (*p* = 0.0014) compared with non‐tumour‐bearing mice (Figure 7b). Representative immunofluorescence images of gastrocnemius are presented in Figure 7c–f for male groups and in Figure 7i–l for female groups. Muscle fibre‐type composition and fibre CSA were also analysed in soleus as an ancillary muscle to gastrocnemius highly recruited during wheel running, and the results/representative images are presented in Figure S2.

## 4 DISCUSSION

Cancer cachexia is a devastating condition, accounting for 20%–40% of cancer‐related deaths (Argiles et al., 2014; Fearon et al., 2011). Exercise training has been suggested as a potential non‐pharmacological therapeutic to counteract tumour‐induced muscle wasting (Courneya et al., 2000; Lira et al., 2014; Tsitkanou et al., 2022). We used an abbreviated progressive weighted wheel‐running model, known as ‘PoWeR’, as a translatable murine model of concurrent resistance and endurance training (Murach et al., 2019; Murach, McCarthy et al., 2020) in tumour‐bearing mice. In this study, surprisingly, PoWeR training itself reduced muscle size regardless of tumour and did not attenuate CC. However, we demonstrated that C26 colorectal tumour‐bearing mice exhibited similar cellular‐ and molecular‐level exercise adaptations to healthy mice in both biological sexes. Specifically, exercise‐induced fibre‐type adaptations in the limb muscle and cardiac hypertrophy were not compromised in tumour‐bearing conditions, even when signs of cancer‐associated dysregulation were present. This finding demonstrates that the ability to adapt normally to exercise is not impaired during CC and provides insight into exercise‐related interventions in cancer patients.

A major unanticipated finding was that PoWeR training in healthy mice did not elicit muscle growth in any of the lower hindlimb muscles and, in fact, decreased soleus and gastrocnemius fibre size. Typically, soleus muscle growth can be seen as early as 4 weeks with PoWeR (Englund et al., 2021) in 3‐month‐old female mice, with the full duration (8 week) PoWeR model yielding ∼10%–20% growth of the soleus and plantaris muscle in mice aged 4–24 months (Dungan et al., 2022; Englund et al., 2021; Murach, Mobley et al., 2020). PoWeR usually does not elicit gastrocnemius whole‐muscle growth regardless of age. Our results also show a further decrease in gastrocnemius (males only) and quadriceps mass in both male and female tumour‐bearing mice. We attribute lower gastrocnemius mass with short‐term PoWeR in this study to the robust fibre‐type transition (i.e., an increased proportion of smaller MyHC IIA fibres, and type IIB fibres becoming smaller in transition), the younger developmental age of mice at the onset of training, when high running volumes might not be ideal, and/or the genetic background used here (BALB/c), whereas previous PoWeR studies typically used adult mice on a C57BL/6J background. Given that reduced muscle mass was observed in exercised healthy mice, we suggest this not to be inherently detrimental, but merely the adaptation to our training modality. There were also imbalances in genes related to muscle protein turnover and inflammatory signalling in gastrocnemius of both males and females. In males, *Redd1* and *Murf1* were relatively more induced in tumour‐bearing PoWeR‐trained mice than in tumour‐bearing sedentary mice. Together, these imbalances in protein turnover might explain, in part, the reduced protein synthesis in males and the lowered muscle mass with both tumour and exercise observed in both biological sexes.

Long‐term (≥8 weeks) moderate‐intensity aerobic exercise training on either a treadmill (Puppa et al., 2012) or a running wheel (Khamoui et al., 2016) can prevent cancer‐induced muscle wasting and weakness without inducing adverse effects in disease progression in the *Apc^Min^* ^/+^ and C26 colorectal cancer mouse models. However, long‐duration exercise training is not always essential to induce beneficial adaptations during CC, on condition that exercise training is mild in volume and/or voluntary. A previous study showed that a short‐term voluntary unweighted wheel running regimen prevented tumour‐induced muscle wasting and dysfunction in C26 mice (Pigna et al., 2016). In our case, significant negative correlations were found between the running distance during the 24 day PoWeR intervention and muscle size in health and/or CC conditions. Several preclinical investigations using concurrent training models during CC show benefit by decelerating disease progression and mitigating muscle wasting (Ballaro et al., 2019; Khamoui et al., 2016; Puppa et al., 2012; Ranjbar et al., 2019; Wood et al., 2022). Conversely, our investigation corroborates that of Pin et al. (2015), who performed 5 days per week of treadmill running in 6‐week‐old mice, which did not preserve muscle mass in tumour‐bearing mice. The commonality of the studies that show benefits is associated with more moderate‐volume exercise training with controlled duration, fixed sets and repetitions, and including rest days (e.g., motorized running wheel and weighted ladder climbing), as opposed to our continuous unmoderated voluntary wheel running. This suggests that high, uncontrolled exercise training volumes might not be optimal to attenuate or prevent juvenile CC and might have implications for exercise prescription in cancer cachexia related to volume, intensity and mode.

Cardiac mass was greater with PoWeR in both biological sexes, regardless of tumour. This observation is congruent with expected physiological cardiac hypertrophy commonly observed with endurance training and promoting cardiac function. Indeed, it was reported previously that the cardiac adaptations to PoWeR in adult mice are similar to an ‘athlete's heart’, with a larger left ventricular cavity volume and lower resting heart rate (Dungan et al., 2019). Although we did not measure cardiac function directly, we speculate that preventing CC‐related cardiac atrophy is likely to yield benefit based on known cardiac adaptations with PoWeR training. This is noteworthy because CC‐induced cardiac atrophy and heart failure are common in cancer patients and represent the primary cause of death in ≤30% of cancer patients (Bordignon et al., 2022; Tichy & Parry, 2023). Thus, short‐term PoWeR training counteracting cardiac cachexia might be a crucial outcome, for which further study is needed.

Biological sexual dimorphism in CC has been described in clinical studies (Zhong & Zimmers, 2020) and preclinical studies (Cabrera et al., 2023; Lim et al., 2022). From a clinical perspective, male cancer patients appear to exhibit a higher prevalence of muscle wasting than female cancer patients (Zhong & Zimmers, 2020). Researchers in our laboratory have shown that LLC‐bearing male mice have many metabolic perturbations preceding the onset of CC (Brown et al., 2017), whereas female LLC mice might maintain muscle myogenic potential, mitochondrial respiratory capacity, mitochondrial dynamics and oxidative capacity during early‐stage development of CC (Lim et al., 2022). Likewise, C26 male mice exhibit early muscle atrophy, whereas female C26 mice appear to exhibit delayed muscle loss during early‐stage cachexia (Cabrera et al., 2023). Biological sexual dimorphism in the response to either exercise (Ansdell et al., 2019, 2020; West et al., 2012) or atrophic stimuli (Rosa‐Caldwell, Lim, Haynie, Brown, Deaver et al., 2021; Rosa‐Caldwell, Lim, Haynie, Brown, Lee et al., 2021; Tsitkanou et al., 2023) has also been well described in otherwise healthy mice. To the best of our knowledge, no study (either preclinical or clinical) has directly investigated biological sex differences in response to exercise in CC in a single study. An indirect and observational comparison between two preclinical studies from the same laboratory, using the same exercise intervention but in different biological sexes, suggests that exercise‐induced adaptations in skeletal muscle are similar in female and male tumour‐bearing mice (Hardee et al., 2016; Sato et al., 2019). Specifically, a translatable murine model of resistance training, including repeated lower‐limb contractions through high‐frequency electrical stimulation for 2 weeks, increases overall muscle weight and the size of type IIA and IIB fibres in the TA muscle, improves myofibrillar protein synthesis and attenuates cachexia‐induced AMPK activity and reduction of skeletal muscle oxidative capacity similarly in female (Sato et al., 2019) and male (Hardee et al., 2016) *Apc^Min^* ^/+^ mice. Interestingly, our PoWeR training here negatively affected only the hindlimbs in female mice, contrary to males, which had lower triceps and pectoralis mass after exercise training. Likewise, Bittel et al. (2024) showed that in mice with facioscapulohumeral muscular dystrophy, 6 weeks of wheel running had a more detrimental impact on the fibre size of triceps in males than females. We also show that male PoWeR‐trained tumour‐bearing mice have reduced running volumes and food consumption in the final weeks of the training. This could explain why only in males was *Pgc1α* more induced in PoWeR‐trained tumour‐bearing mice than in sedentary tumour‐bearing mice, given the well‐known role of *Pgc1α* as a cellular energy sensor (Canto & Auwerx, 2009). Further investigation is needed to better define biological sex divergence in response to exercise during CC, including both subcutaneous and orthotopic CC mouse models, in addition to different exercise modes and intensities.

PoWeR training increased the mRNA content of *Pgc1α*, a primary regulator of oxidative adaptations, in gastrocnemius of both males and females. Higher *Pgc1α* mRNA levels with PoWeR regardless of tumour is consistent with a shift towards a more oxidative fibre‐type profile, in which this molecule is mechanistically implicated (Lin et al., 2002; Rasbach et al., 2010). However, only in males was the exercise‐induced *Pgc1α* increase statistically significant between exercised tumour‐bearing mice and sedentary tumour‐bearing mice, suggesting a potentially more robust mitochondrial response in the tumour‐bearing state in males compared with females. Considering that females are more fatigue resistant and exhibit enhanced mitochondrial quality and maintenance compared with male muscles (Ansdell et al., 2020; Brown et al., 2017; Lim et al., 2022; Rosa‐Caldwell & Greene, 2019; Rosa‐Caldwell, Lim, Haynie, Brown, Lee et al., 2021), a greater exercise workload in the PoWeR protocol (longer exercise training period or heavier resistance) might be required for females to present similar changes in oxidative muscle characteristics to males. Finally, although we acknowledge the limitation of our study in not measuring the protein levels of transcriptional responses, including *Pgc1α* transcription, assessing mRNA levels could be considered equally reliable because of the poor specificity of antibodies and the very short half‐life of target proteins in this study.

## 5 CONCLUSION

In conclusion, short‐term PoWeR training during the symptomatic stage of CC (i.e., after the inoculation of C26 cells) induces similar skeletal muscle adaptations in males and females. This suggests that the plasticity and ability of the muscles to adapt to exercise is preserved during early‐stage cachexia. However, our model of high‐volume concurrent exercise training induces muscle loss, which might be confounded by the young developmental age and/or strain of mice used in this investigation. At the molecular level, protein turnover deficiencies in skeletal muscle in both biological sexes translated to decreased protein synthesis only in males. Forelimb muscle mass, running volume and food consumption were maintained in females throughout tumour development, whereas these variables decreased in males. Despite exercise‐induced muscle atrophy and molecular/protein synthesis dysregulations in male mice, both biological sexes presented training adaptations including a faster‐to‐slower fibre‐type transition in gastrocnemius and an increase in heart mass. Performing PoWeR training for only 24 days appears to be sufficient to induce remodelling of skeletal muscle associated with the endurance aspect of the training. The effects of prolonging the exercise training period and/or adjusting (increasing or decreasing) the resistance of wheel running need to be investigated further in both biological sexes.

## AUTHOR CONTRIBUTIONS

The manuscript was written by Stavroula Tsitkanou. Experimental design and analyses were conceived by Nicholas P. Greene, Stavroula Tsitkanou, Kevin A. Murach and Pieter Koopmans. Animal experiments were performed by Stavroula Tsitkanou and Pieter Koopmans. Analyses were performed by Stavroula Tsitkanou, Pieter Koopmans, Calvin Peterson, Ana R. Cabrera, Ruqaiza Muhyudin, Francielly Morena, Sabin Khadgi and Eleanor R. Schrems. Nicholas P. Greene and Kevin A. Murach supervised and obtained funding for this work. All authors contributed intellectually, provided feedback, approved the final version of the manuscript and agree to be accountable for all aspects of the work in ensuring that questions related to the accuracy or integrity of any part of the work are appropriately investigated and resolved. All persons designated as authors qualify for authorship, and all those who qualify for authorship are listed.

## CONFLICT OF INTEREST

No conflicts of interest, financial or otherwise, are declared by the authors.

## Supporting information




**FIGURE S1** Unnormalized data of body weight (in grams) during cancer cachexia progression in males (a) and females (b). https://figshare.com/s/0e9f377cf1052a26ced5

**FIGURE S2** In soleus, PoWeR exercise training induces muscle fibre adaptations only in males and not in females. Exercised males have lower cross‐sectional area (CSA) in myosin heavy chain (MyHC) type I (a), a higher percentage of MyHC I (b), a lower percentage of MyHC IIA (b), more central myonuclei per fibre (c) and greater spindle size (d). Tumour‐bearing males have lower CSA of MyHC IIA (a). In female mice, neither exercise nor tumour affects muscle fibre size (i) and composition (j), number of central myonuclei per fibre (k) and spindle size (l). A red asterisk symbolizes a significant (*p* < 0.05) tumour main effect (C26 vs. PBS), and a blue asterisk symbolizes a significant (*p* < 0.05) PoWeR exercise main effect (PoWeR vs. SED). Immunofluorescence images (×20 magnification) are presented for male groups (e–h) and for female groups (m–p). MyHC I fibres are stained with pink, MyHC IIA fibres are unstained (‘black’ fibres), sarcolemma is stained with red and myonuclei with blue. White arrow (e) indicates a muscle spindle. https://figshare.com/s/76ad6def0e3febdb6722

**FIGURE S3** A significant main effect of biological sex (pink asterisk) and condition (brown asterisk) was observed in tumour‐free body weight, expressed as the percentage change from baseline (day 0 of C26 inoculation) to the end‐point (a), using a two‐way ANOVA between biological sexes. Additionally, a significant interaction between biological sex and condition was found in tumour‐free body weight (*p* = 0.0006), with Tukey's *post hoc* comparisons presented in the upper right table (a). A main effect of biological sex (pink asterisk) was also found in the fractional synthetic rate (FSR), expressed as the percentage change relative to same‐sex healthy controls (b). Moreover, a significant interaction between biological sex and condition was observed (*p* = 0.049) in FSR, with Tukey's *post hoc* comparisons shown in the bottom right table (b). https://figshare.com/s/a067c4349c1ce6900808

**FIGURE S4** Biological sex comparisons were conducted for running distance (a) and food consumption (b) over the intervention period, expressed as the area under the curve (AUC), using a two‐way ANOVA. A significant main effect of biological sex (pink asterisk) and condition (brown asterisk) was observed in running distance, with no significant interaction. Additionally, a biological sex comparison was performed for food consumption, expressed as the percentage change from the first week of exercise training to the end‐point (c), using Student's unpaired *t*‐test between male and female exercised tumour‐bearing mice (C26 PoWeR). Females maintained food consumption, whereas males exhibited a substantial decline compared with females (c). The black asterisk (c) denotes a significant difference (*p* < 0.05) between males and females, as determined by Student's unpaired *t*‐test. https://figshare.com/s/a61a9bb5c50a78bfe657

**TABLE S1** Tissue weights (mean ± SD) expressed as percentage change from the sedentary non‐tumour‐bearing mice (PBS SED) in males and females. A main effect of biological sex (*p* ≤ 0.0001) was found in triceps, pectoralis and liver, and a main effect of condition (*p* < 0.0001–0.037) was found in plantaris, gastrocnemius, quadriceps, triceps, pectoralis, heart, spleen and gonadal fat using a two (males and females) by three (PBS PoWeR, C26 SED and C26 PoWeR) ANOVA. https://figshare.com/s/f2a987d6a6fef8f8bd3f

**TABLE S2** Pearson's *r* and *p*‐values of the correlations between running distance, expressed as area under the curve (AUC), and tumour‐free body weight (BW), normalized weight (by tibia length) of soleus, plantaris, gastrocnemius, tibialis anterior (TA), extensor digitorum longus (EDL), quadriceps, triceps, pectoralis, heart, liver, spleen and gonadal fat, in addition to the tumour weight at the experimental end‐point, pooling male and female: (i) non‐tumour‐ and tumour‐bearing mice (*n* = 33); (ii) only tumour‐bearing mice (*n* = 15); and (iii) only non‐tumour‐bearing mice (*n* = 18). Red *p*‐values represent a statistically significant correlation (*p* < 0.05). https://figshare.com/s/cc120f0f9e8b67df4bd0

## Data Availability

Data will be made available upon reasonable request to the corresponding author(s).
