# Supplementary figures and images for: Myocellular adaptations to short‐term weighted wheel‐running exercise are largely conserved during C26‐tumour induction in male and female mice
Source: Exp Physiol. 2025 Apr 24;111(6):3039–54. doi: 10.1113/EP092504 (PMC13238660; doi:10.1113/EP092504)

(a)

**Males**

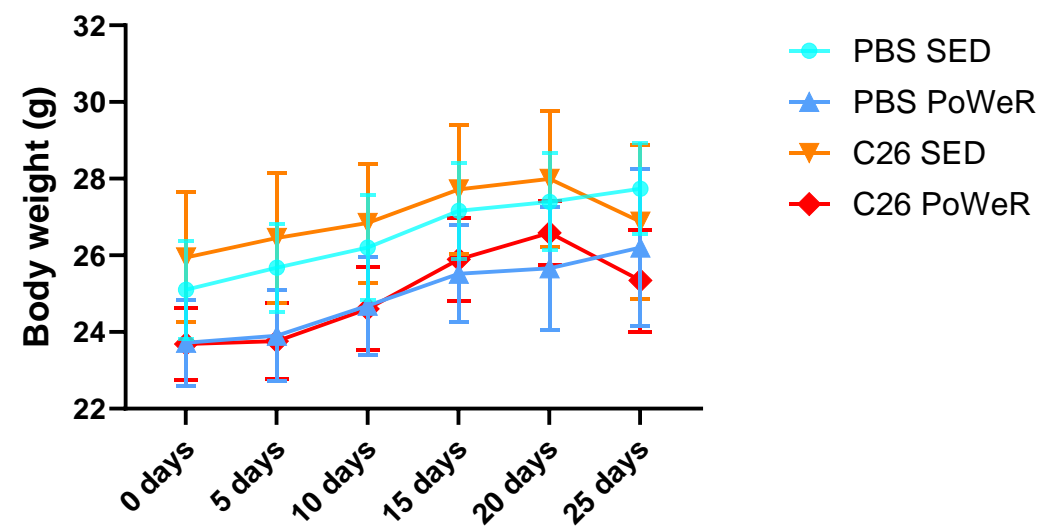

(b)

**Females**

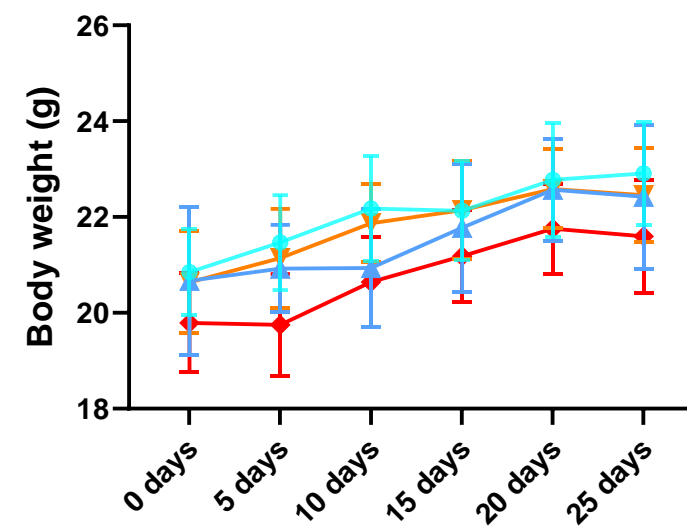

Supplement: Supplementary file 1 — FIGURE S1 Unnormalized data of body weight (in grams) during cancer cachexia progression in males (a) and females (b). https://figshare.com/s/0e9f377cf1052a26ced5 [file EPH-111-3039-s003.pdf]

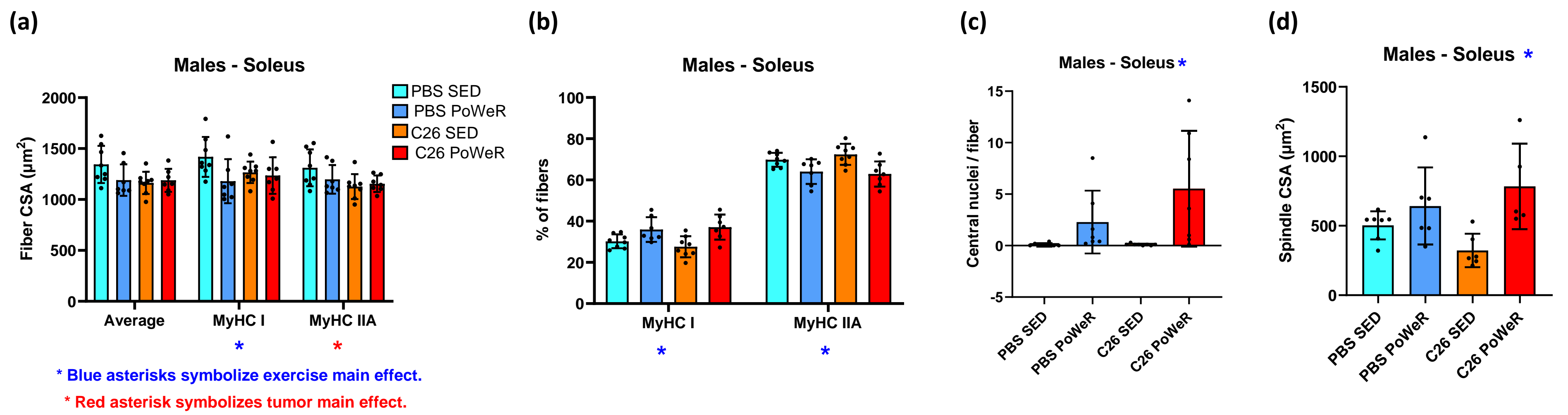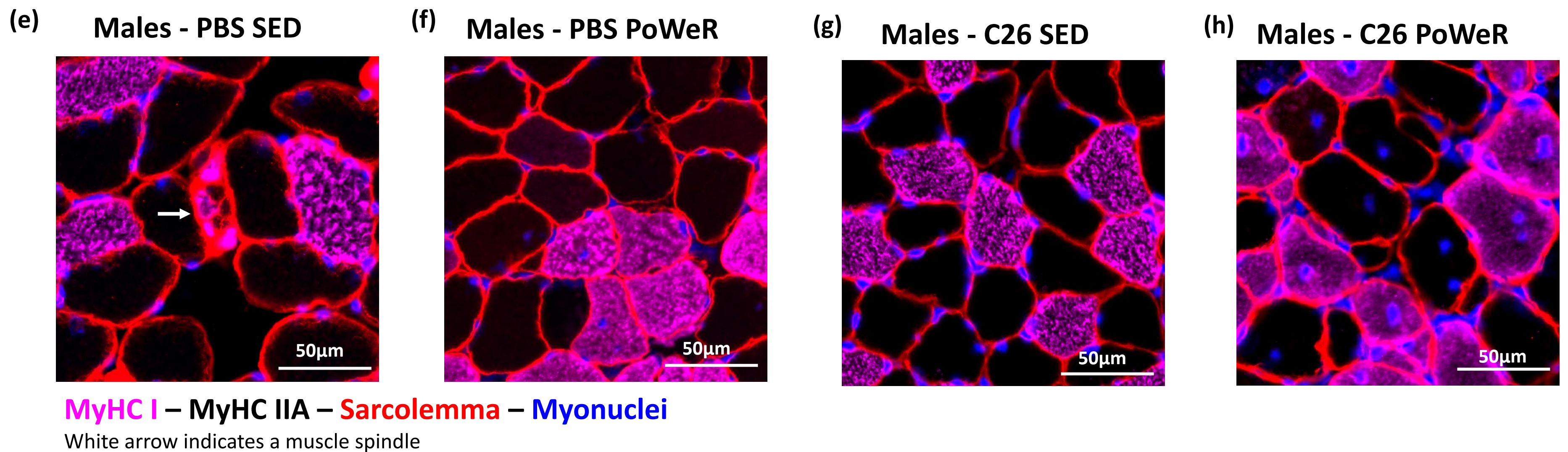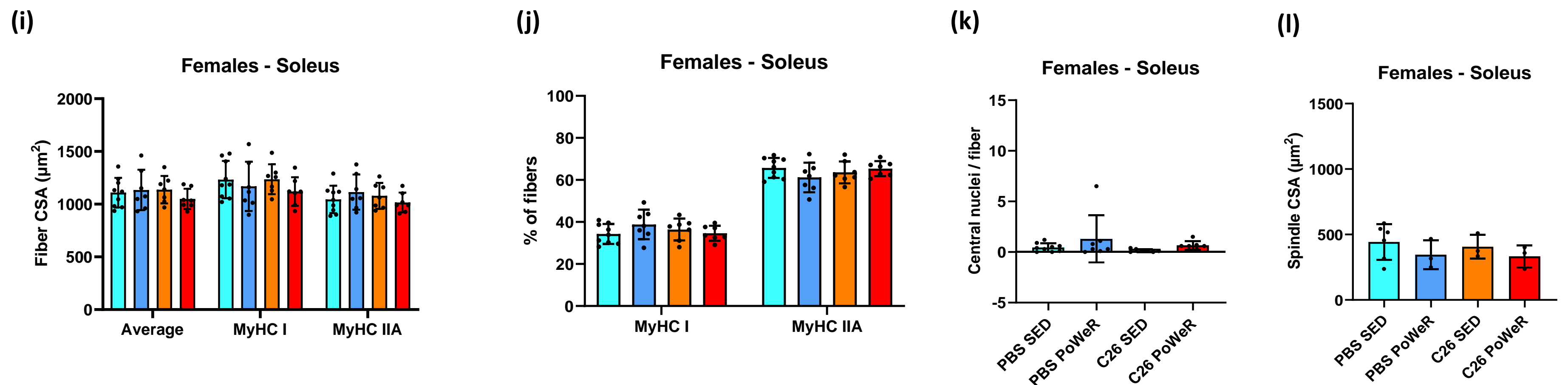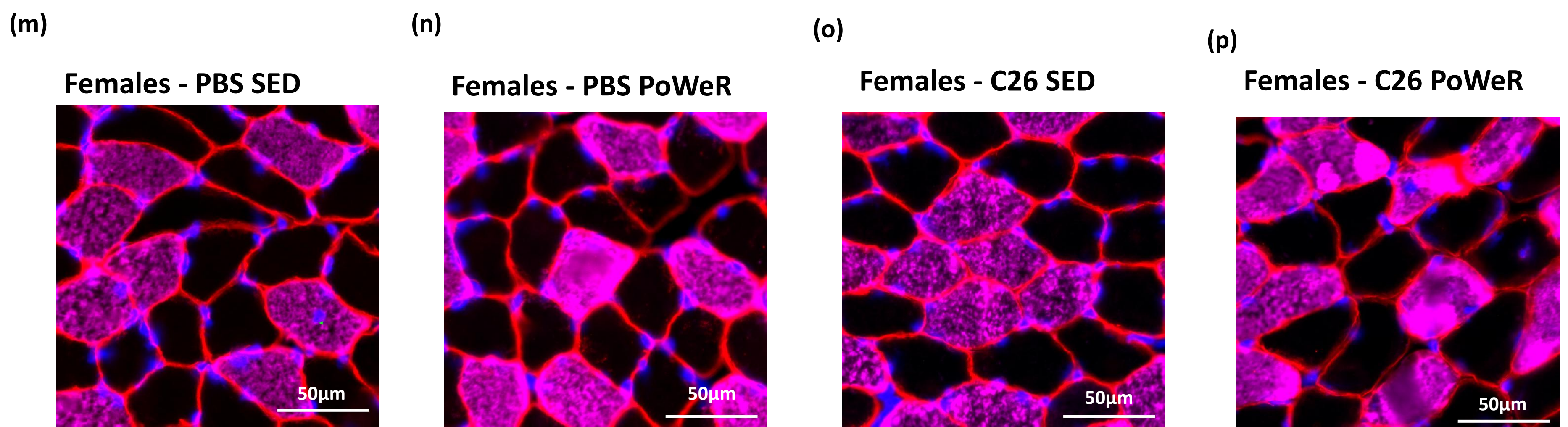

Supplement: Supplementary file 2 — FIGURE S2 In soleus, PoWeR exercise training induces muscle fibre adaptations only in males and not in females. Exercised males have lower cross‐sectional area (CSA) in myosin heavy chain (MyHC) type I (a), a higher percentage of MyHC I (b), a lower percentage of MyHC IIA (b), more central myonuclei per fibre (c) and greater spindle size (d). Tumour‐bearing males have lower CSA of MyHC IIA (a). In female mice, neither exercise nor tumour affects muscle fibre size (i) and composition (j), number of central myonuclei per fibre (k) and spindle size (l). A red asterisk symbolizes a significant (p < 0.05) tumour main effect (C26 vs. PBS), and a blue asterisk symbolizes a significant (p < 0.05) PoWeR exercise main effect (PoWeR vs. SED). Immunofluorescence images (×20 magnification) are presented for male groups (e–h) and for female groups (m–p). MyHC I fibres are stained with pink, MyHC IIA fibres are unstained (‘black’ fibres), sarcolemma is stained with red and myonuclei with blue. White arrow (e) indicates a muscle spindle. https://figshare.com/s/76ad6def0e3febdb6722 [file EPH-111-3039-s002.pdf]
