# Supplementary material for: Myocellular adaptations to short‐term weighted wheel‐running exercise are largely conserved during C26‐tumour induction in male and female mice
Source: Exp Physiol. 2025 Apr 24;111(6):3039–54. doi: 10.1113/EP092504 (PMC13238660; doi:10.1113/EP092504)
Supplement: Supplementary file 3 — FIGURE S3 A significant main effect of biological sex (pink asterisk) and condition (brown asterisk) was observed in tumour‐free body weight, expressed as the percentage change from baseline (day 0 of C26 inoculation) to the end‐point (a), using a two‐way ANOVA between biological sexes. Additionally, a significant interaction between biological sex and condition was found in tumour‐free body weight (p = 0.0006), with Tukey's post hoc comparisons presented in the upper right table (a). A main effect of biological sex (pink asterisk) was also found in the fractional synthetic rate (FSR), expressed as the percentage change relative to same‐sex healthy controls (b). Moreover, a significant interaction between biological sex and condition was observed (p = 0.049) in FSR, with Tukey's post hoc comparisons shown in the bottom right table (b). https://figshare.com/s/a067c4349c1ce6900808 [file EPH-111-3039-s006.pdf]

(a)

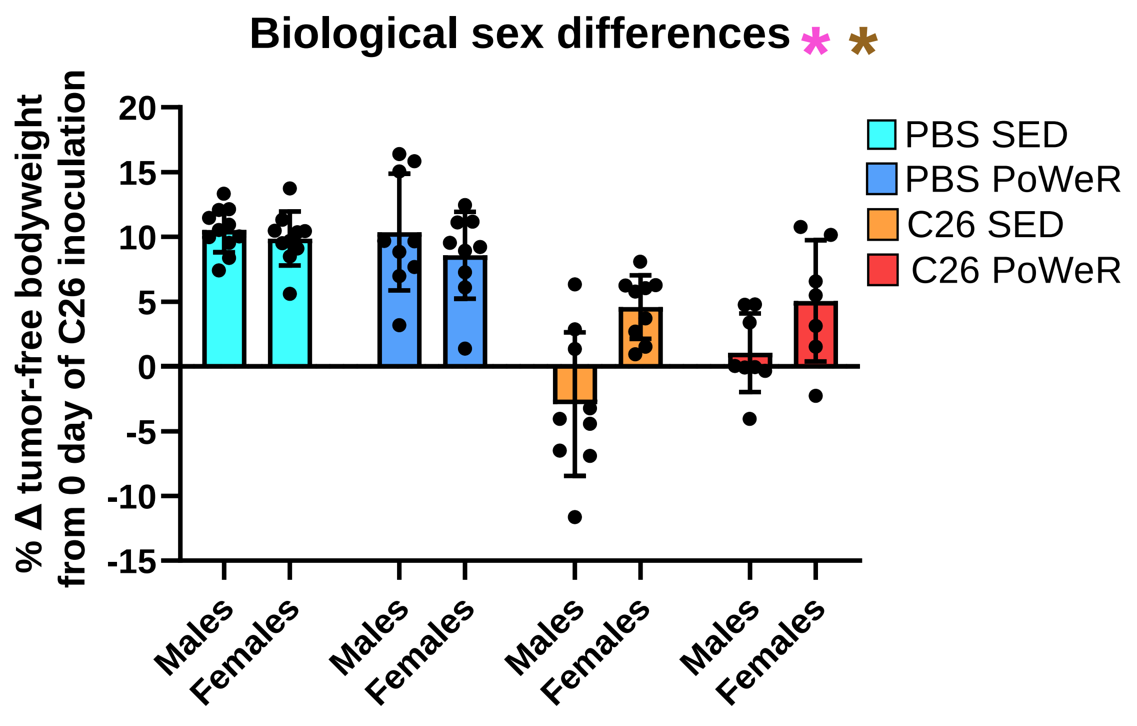

* Pink asterisk represents biological sex main effect

* Brown asterisk represents condition main effect

Interaction: p=0.0006

% Δ tumor-free bodyweight from 0 day of C26 inoculation			
	Tukey's post hoc analysis (Interaction: Sex x Condition, p=0.0006)	Mean difference	p value
Comparisons within males	Males:PBS SED vs. Males:PBS PoWeR	0.1613	>0.9999
	Males:PBS SED vs. Males:C26 SED	13.44	<0.0001
	Males:PBS SED vs. Males:C26 PoWeR	9.47	<0.0001
	Males:PBS PoWeR vs. Males:C26 SED	13.28	<0.0001
	Males:PBS PoWeR vs. Males:C26 PoWeR	9.309	<0.0001
	Males:C26 SED vs. Males:C26 PoWeR	-3.97	0.312
Comparisons within females	Females:PBS SED vs. Females:PBS PoWeR	1.297	0.9929
	Females:PBS SED vs. Females:C26 SED	5.286	0.0384
	Females:PBS SED vs. Females:C26 PoWeR	4.82	0.1272
	Females:PBS PoWeR vs. Females:C26 SED	3.989	0.2699
	Females:PBS PoWeR vs. Females:C26 PoWeR	3.522	0.5136
	Females:C26 SED vs. Females:C26 PoWeR	-0.4665	>0.9999
Comparisons between Males vs. Females	Males:PBS SED vs. Females:PBS SED	0.6551	0.9999
	Males:PBS SED vs. Females:PBS PoWeR	1.952	0.9228
	Males:PBS SED vs. Females:C26 SED	5.941	0.0096
	Males:PBS SED vs. Females:C26 PoWeR	5.475	0.044
	Males:PBS PoWeR vs. Females:PBS SED	0.4938	>0.9999
	Males:PBS PoWeR vs. Females:PBS PoWeR	1.791	0.9612
	Males:PBS PoWeR vs. Females:C26 SED	5.78	0.0213
	Males:PBS PoWeR vs. Females:C26 PoWeR	5.313	0.0769
	Males:C26 SED vs. Females:PBS SED	-12.79	<0.0001
	Males:C26 SED vs. Females:PBS PoWeR	-11.49	<0.0001
	Males:C26 SED vs. Females:C26 SED	-7.499	0.0008
	Males:C26 SED vs. Females:C26 PoWeR	-7.965	0.0009
	Males:C26 PoWeR vs. Females:PBS SED	-8.815	<0.0001
	Males:C26 PoWeR vs. Females:PBS PoWeR	-7.518	0.0012
	Males:C26 PoWeR vs. Females:C26 SED	-3.529	0.4635
	Males:C26 PoWeR vs. Females:C26 PoWeR	-3.996	0.3837

(b)

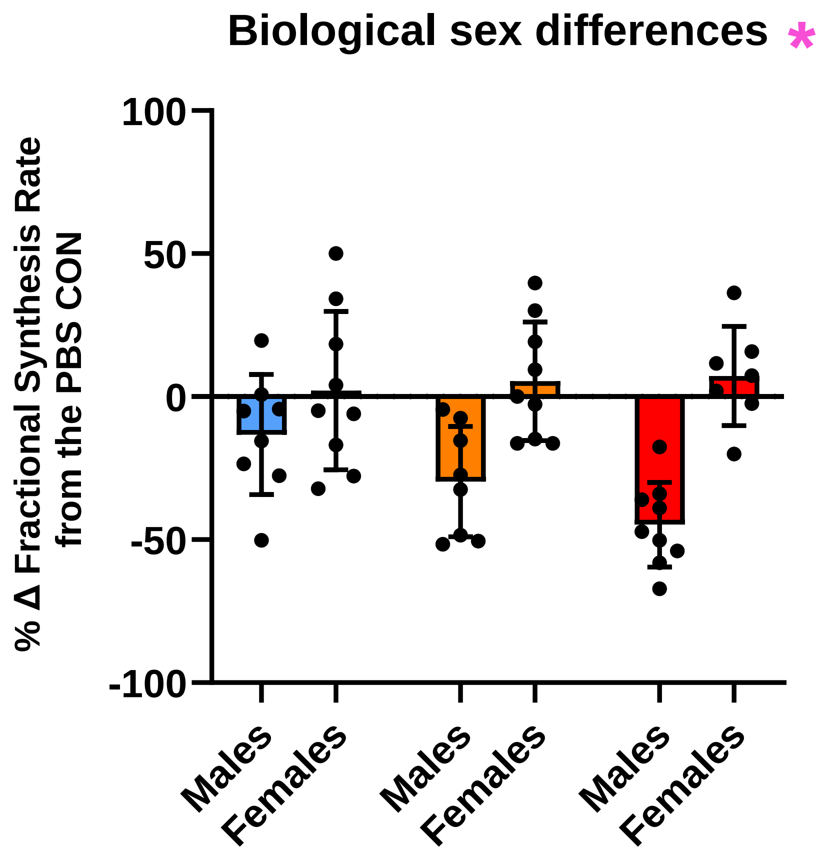

* Pink asterisk represents biological sex main effect

Interaction: p=0.049

% Δ Fractional Synthesis Rate from the PBS CON			
	Tukey's post hoc analysis (Interaction: Sex x Condition, p=0.049)	Mean difference	p value
Comparisons within males	Males:PBS PoWeR vs. Males:C26 CON	16.49	0.6077
	Males:PBS PoWeR vs. Males:C26 PoWeR	31.53	0.034
	Males:C26 CON vs. Males:C26 PoWeR	15.05	0.6688
Comparisons within females	Females:PBS PoWeR vs. Females:C26 CON	-3.264	0.9994
	Females:PBS PoWeR vs. Females:C26 PoWeR	-5.134	0.9962
	Females:C26 CON vs. Females:C26 PoWeR	-1.87	>0.9999
Comparisons between Males vs. Females	Males:PBS PoWeR vs. Females:PBS PoWeR	-15.34	0.6507
	Males:PBS PoWeR vs. Females:C26 CON	-18.6	0.4462
	Males:PBS PoWeR vs. Females:C26 PoWeR	-20.47	0.4096
	Males:C26 CON vs. Females:PBS PoWeR	-31.83	0.0316
	Males:C26 CON vs. Females:C26 CON	-35.09	0.0134
	Males:C26 CON vs. Females:C26 PoWeR	-36.96	0.0149
	Males:C26 PoWeR vs. Females:PBS PoWeR	-46.87	0.0003
	Males:C26 PoWeR vs. Females:C26 CON	-50.14	<0.0001
	Males:C26 PoWeR vs. Females:C26 PoWeR	-52.01	0.0001
