# Supplementary material for: Myocellular adaptations to short‐term weighted wheel‐running exercise are largely conserved during C26‐tumour induction in male and female mice
Source: Exp Physiol. 2025 Apr 24;111(6):3039–54. doi: 10.1113/EP092504 (PMC13238660; doi:10.1113/EP092504)
Supplement: Supplementary file 4 — FIGURE S4 Biological sex comparisons were conducted for running distance (a) and food consumption (b) over the intervention period, expressed as the area under the curve (AUC), using a two‐way ANOVA. A significant main effect of biological sex (pink asterisk) and condition (brown asterisk) was observed in running distance, with no significant interaction. Additionally, a biological sex comparison was performed for food consumption, expressed as the percentage change from the first week of exercise training to the end‐point (c), using Student's unpaired t‐test between male and female exercised tumour‐bearing mice (C26 PoWeR). Females maintained food consumption, whereas males exhibited a substantial decline compared with females (c). The black asterisk (c) denotes a significant difference (p < 0.05) between males and females, as determined by Student's unpaired t‐test. https://figshare.com/s/a61a9bb5c50a78bfe657 [file EPH-111-3039-s001.pdf]

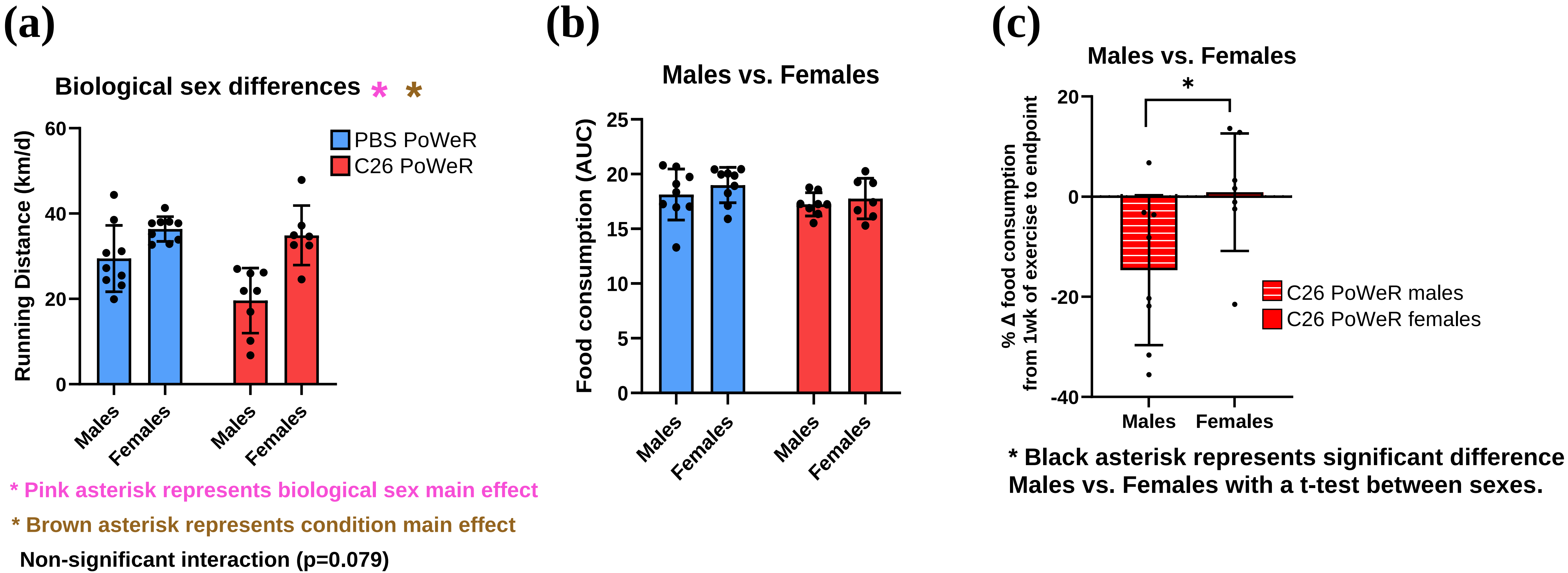
(a)
Biological sex differences * *
Running Distance (km/d)
60
40
20
0
PBS PoWeR
C26 PoWeR
Males
Females
Males
Females
* Pink asterisk represents biological sex main effect
* Brown asterisk represents condition main effect
Non-significant interaction (p=0.079)
(b)
Males vs. Females
Food consumption (AUC)
25
20
15
10
5
0
Males
Females
Males
Females
(c)
Males vs. Females
*
% Δ food consumption from 1wk of exercise to endpoint
20
0
-20
-40
C26 PoWeR males
C26 PoWeR females
Males
Females
* Black asterisk represents significant difference Males vs. Females with a t-test between sexes.
