# Supplementary material for: Myocellular adaptations to short‐term weighted wheel‐running exercise are largely conserved during C26‐tumour induction in male and female mice
Source: Exp Physiol. 2025 Apr 24;111(6):3039–54. doi: 10.1113/EP092504 (PMC13238660; doi:10.1113/EP092504)
Supplement: Supplementary file 5 — TABLE S1 Tissue weights (mean ± SD) expressed as percentage change from the sedentary non‐tumour‐bearing mice (PBS SED) in males and females. A main effect of biological sex (p ≤ 0.0001) was found in triceps, pectoralis and liver, and a main effect of condition (p < 0.0001–0.037) was found in plantaris, gastrocnemius, quadriceps, triceps, pectoralis, heart, spleen and gonadal fat using a two (males and females) by three (PBS PoWeR, C26 SED and C26 PoWeR) ANOVA. https://figshare.com/s/f2a987d6a6fef8f8bd3f [file EPH-111-3039-s005.docx]

**Supplemental Table 1.** Tissue weights (mean ± SD) expressed as percentage change from the sedentary non‐tumour‐bearing mice (PBS SED) in males and females. A main effect of biological sex (p ≤ 0.0001) was found in triceps, pectoralis and liver, and a main effect of condition (p < 0.0001–0.037) was found in plantaris, gastrocnemius, quadriceps, triceps, pectoralis, heart, spleen and gonadal fat using a two (males and females) by three (PBS PoWeR, C26 SED and C26 PoWeR) ANOVA.

|  | % change from PBS SED in MALES | | | % change from PBS SED in FEMALES | | | Biological Sex Main Effect | Condition Main Effect | Interaction |
| --- | --- | --- | --- | --- | --- | --- | --- | --- | --- |
| Tissue | **PBS PoWeR (n=9)** | **C26 SED (n=9)** | **C26 PoWeR (n=8)** | **PBS PoWeR (n=9)** | **C26 SED (n=9)** | **C26 PoWeR (n=7)** | **p value** | **p value** | **p value** |
| Soleus | -2.27 ± 14.32 | -5.46 ± 8.11 | 0.53 ± 12.84 | 4.50 ± 6.51 | -3.51 ± 8.92 | -0.01 ± 7.17 | 0.343 | 0.218 | 0.573 |
| Plantaris | -5.75 ± 7.76 | -8.98 ± 8.54 | -10.02 ± 4.78 | 1.96 ± 8.23 | -11.73 ± 10.04 | -5.99 ± 5.55 | 0.181 | **0.007** | 0.140 |
| Gastrocnemius | -8.51 ± 6.02 | -6.90 ± 6.95 | -14.12 ± 4.14 | -5.67 ± 6.17 | -4.68 ± 6.06 | -10.25 ± 4.37 | 0.074 | **0.008** | 0.920 |
| TA | -6.73 ± 4.76 | -7.80 ± 10.15 | -11.96 ± 8.08 | -3.34 ± 5.57 | -3.33 ± 5.70 | -8.30 ± 5.93 | 0.056 | 0.088 | 0.972 |
| EDL | -10.37 ± 8.85 | -1.49 ± 11.08 | -3.14 ± 14.42 | -2.94 ± 10.62 | -6.36 ± 9.39 | -11.48 ± 6.34 | 0.516 | 0.607 | 0.080 |
| Quadriceps | -7.33 ± 4.01 | -3.81 ± 8.51 | -18.54 ± 9.14 | -2.44 ± 5.00 | -2.49 ± 8.38 | -16.20 ± 8.96 | 0.184 | **<0.0001** | 0.767 |
| Triceps | -9.72 ± 8.55 | -7.83 ± 5.06 | -13.95 ± 4.70 | 0.90 ± 8.13 | -0.56 ± 7.84 | -6.31 ± 7.16 | **0.0001** | **0.037** | 0.746 |
| Pectoralis | -18.78 ± 12.18 | -9.27 ± 16.42 | -22.34 ± 11.64 | -2.34 ± 13.71 | 5.09 ± 13.67 | -5.15 ± 8.31 | **<0.0001** | **0.036** | 0.948 |
| Heart | 12.54 ± 8.97 | -4.65 ± 7.21 | 1.93 ± 8.22 | 9.39 ± 9.28 | -3.75 ± 6.86 | 0.92 ± 4.72 | 0.622 | **<0.0001** | 0.758 |
| Liver | 0.46 ± 11.61 | -2.00 ± 7.90 | -4.91 ± 6.99 | 14.74 ± 11.88 | 6.75 ± 6.51 | 13.55 ± 10.02 | **<0.0001** | 0.254 | 0.339 |
| Spleen | -6.88 ± 12.01 | 115.63 ± 38.34 | 90.95 ± 17.27 | 1.26 ± 6.53 | 99.53 ± 32.30 | 70.96 ± 30.08 | 0.200 | **<0.0001** | 0.225 |
| Fat | -43.78 ± 10.54 | -22.50 ± 23.03 | -71.95 ± 10.51 | -41.70 ± 16.91 | -29.31 ± 33.47 | -72.59 ± 8.04 | 0.749 | **<0.0001** | 0.789 |

TA, tibialis anterior and EDL, extensor digitorum longus.
