# Supplementary material for: Myocellular adaptations to short‐term weighted wheel‐running exercise are largely conserved during C26‐tumour induction in male and female mice
Source: Exp Physiol. 2025 Apr 24;111(6):3039–54. doi: 10.1113/EP092504 (PMC13238660; doi:10.1113/EP092504)
Supplement: Supplementary file 6 — TABLE S2 Pearson's r and p‐values of the correlations between running distance, expressed as area under the curve (AUC), and tumour‐free body weight (BW), normalized weight (by tibia length) of soleus, plantaris, gastrocnemius, tibialis anterior (TA), extensor digitorum longus (EDL), quadriceps, triceps, pectoralis, heart, liver, spleen and gonadal fat, in addition to the tumour weight at the experimental end‐point, pooling male and female: (i) non‐tumour‐ and tumour‐bearing mice (n = 33); (ii) only tumour‐bearing mice (n = 15); and (iii) only non‐tumour‐bearing mice (n = 18). Red p‐values represent a statistically significant correlation (p < 0.05). https://figshare.com/s/cc120f0f9e8b67df4bd0 [file EPH-111-3039-s004.docx]

**Supplemental table 2.** Correlations between running distance, expressed as area under the curve (AUC), and tumour-free body weight (BW), tumour and normalized tissue weights (by tibia length).

|  |  | Correlation with running distance (AUC) | |
| --- | --- | --- | --- |
|  |  | Pearson’s r | p value |
| Both C26 and PBS mice (n = 33) | **Tumour-free BW** | -0.3825 | 0.028 |
|  | **Soleus** | -0.343 | 0.0507 |
|  | **Plantaris** | -0.4499 | 0.0086 |
|  | **Gastrocnemius** | -0.5389 | 0.0012 |
|  | **TA** | -0.1039 | 0.565 |
|  | **EDL** | -0.4134 | 0.0168 |
|  | **Quadriceps** | -0.3196 | 0.0698 |
|  | **Triceps** | -0.5061 | 0.0027 |
|  | **Pectoralis** | -0.6141 | 0.0001 |
|  | **Heart** | 0.1268 | 0.4819 |
|  | **Liver** | -0.1543 | 0.3913 |
|  | **Spleen** | -0.357 | 0.0414 |
|  | **Fat** | -0.1227 | 0.4962 |
|  | **Tumour** | -0.5898 | 0.0206 |
| Only C26 mice (n = 15) | **Tumour-free BW** | -0.6107 | 0.0156 |
|  | **Soleus** | -0.4077 | 0.1315 |
|  | **Plantaris** | -0.6561 | 0.0079 |
|  | **Gastrocnemius** | -0.7806 | 0.0006 |
|  | **TA** | -0.176 | 0.5305 |
|  | **EDL** | -0.5219 | 0.046 |
|  | **Quadriceps** | -0.6852 | 0.0048 |
|  | **Triceps** | -0.867 | <0.0001 |
|  | **Pectoralis** | -0.8124 | 0.0002 |
|  | **Heart** | -0.1657 | 0.555 |
|  | **Liver** | -0.1279 | 0.6498 |
|  | **Spleen** | -0.1783 | 0.5248 |
|  | **Fat** | -0.745 | 0.0014 |
|  | **Tumour** | -0.5898 | 0.0206 |
| Only PBS mice (n = 18) | **Tumour-free BW** | -0.6343 | 0.0047 |
|  | **Soleus** | -0.3138 | 0.2047 |
|  | **Plantaris** | -0.5172 | 0.028 |
|  | **Gastrocnemius** | -0.6014 | 0.0083 |
|  | **TA** | -0.3555 | 0.1477 |
|  | **EDL** | -0.2848 | 0.252 |
|  | **Quadriceps** | -0.4488 | 0.0618 |
|  | **Triceps** | -0.3638 | 0.1378 |
|  | **Pectoralis** | -0.6036 | 0.008 |
|  | **Heart** | 0.06529 | 0.7969 |
|  | **Liver** | -0.3523 | 0.1516 |
|  | **Spleen** | 0.06445 | 0.7995 |
|  | **Fat** | -0.3513 | 0.1529 |
